# Chronic *Salmonella* Typhi carriage at sites other than the gallbladder

**DOI:** 10.1371/journal.pntd.0011168

**Published:** 2023-03-23

**Authors:** Seth A. Hoffman, Michael J. Sikorski, Myron M. Levine

**Affiliations:** 1 Center for Vaccine Development and Global Health, University of Maryland School of Medicine, Baltimore, Maryland, United States of America; 2 Department of Medicine, University of Maryland School of Medicine, Baltimore, Maryland, United States of America; 3 Institute for Genome Sciences, University of Maryland School of Medicine, Baltimore, Maryland, United States of America; 4 Department of Microbiology and Immunology, University of Maryland School of Medicine, Baltimore, Maryland, United States of America; 5 Department of Pediatrics, University of Maryland School of Medicine, Baltimore, Maryland, United States of America; Yale University School of Medicine, UNITED STATES

## Abstract

Typhoid fever caused by infection with *Salmonella enterica* subspecies *enterica* serotype Typhi (*S*. Typhi), an important public health problem in many low- and middle-income countries, is transmitted by ingestion of water or food contaminated by feces or urine from individuals with acute or chronic *S*. Typhi infection. Most chronic *S*. Typhi carriers (shedding for ≥12 months) harbor infection in their gallbladder wherein preexisting pathologies, particularly cholelithiasis, provide an environment that fosters persistence. Much less appreciated is the existence of non-gallbladder hepatobiliary chronic *S*. Typhi carriers and urinary carriers. The former includes parasitic liver flukes as a chronic carriage risk factor. Chronic urinary carriers typically have pathology of their urinary tract, with or without renal or bladder stones. Even as the prevalence of multidrug-resistant and extensively drug-resistant *S*. Typhi strains is rising, global implementation of highly effective typhoid vaccines is increasing. There is also renewed interest in identifying, monitoring, and (where possible) treating chronic carriers who comprise the long-term reservoir of *S*. Typhi.

## Introduction

Typhoid fever, the human host-restricted infection caused by *Salmonella enterica* subspecies *enterica* serotype Typhi (*S*. Typhi), remains a cardinal health burden for residents of many low- and middle-income countries (LMICs) who lack potable water and improved sanitation. Residents of high-income countries (HICs) mainly acquire typhoid fever by travel to typhoid-endemic LMICs and occasionally by transmission from chronic carriers residing in HICs (including immigrants from typhoid-endemic countries). During acute typhoid infection, *S*. Typhi that are shed in the feces and urine of the infected individual may lead to transmission to proximal contacts (“short cycle transmission”) via contamination of food during preparation or handling by temporary or chronic carriers with improper hygiene practices [1–3]; “long cycle transmission” mainly ensues via consumption of water sources contaminated with human feces or by crops irrigated with untreated sewage [4,5].

In the pathogenesis of acute *S*. Typhi infection, ingested bacilli that survive gastric transit translocate from the lumen of the small intestine (mainly via M cells overlying gut-associated lymphoid tissue) to reach the mucosal lamina propria [6], enter the lymphatic drainage system, and access the blood circulation via the thoracic duct [7,8]. During this primary bacteremia [9], when typhoid bacilli are filtered from the circulation by mononuclear phagocytic cells of the spleen, liver, and bone marrow [7,8], they also reach the gallbladder hematogenously at that point or shortly thereafter by infected hepatic bile entering the gallbladder [7]. After an incubation period of approximately 8 to 14 days, the acute stage of clinical illness commences, characterized by a low-level secondary bacteremia [10,11]. Bile and bile-containing duodenal fluid yield *S*. Typhi at this stage, with the latter providing a useful clinical specimen for diagnostic purposes [12]. Early investigators reported that clinical specimens besides blood and stool can also serve to isolate *S*. Typhi from patients with acute typhoid fever including bile, urine, and skin snips of the evanescent rose spots [13]. Decades later, bone marrow, if obtainable, was ultimately confirmed as the gold standard clinical specimen [14].

Soon after the initial report of isolation of typhoid bacilli in the pre-antibiotic era [15], studies revealed that patients with acute typhoid and convalescents excrete the pathogen in their stools, and some contacts of typhoid cases shed the pathogen asymptomatically [16]. To control typhoid in endemic areas of Southwest Germany, Robert Koch established “Bacteriological Stations” to identify the source of infection (typhoid fever cases) and to render them innocuous by disinfecting their excreta [17]. Koch’s teams looked for unhygienic conditions during household visits (e.g., human feces used for fertilizer, unkempt privies) and repeatedly examined stools and urine of convalescents to determine when they ceased shedding typhoid bacilli, to reduce the risk of them transmitting the disease [17]. Less compliant cases were sent to a hospital to isolate them until their stool excretion ceased, while more compliant patients were followed in their domiciles [17]. Investigators in other countries followed Koch’s example and undertook to monitor the duration of *S*. Typhi excretion in stools by convalescents (reviewed by Ledingham and Arkwright) [18]. They found that a small percentage (2% to 5%) of individuals excrete typhoid bacilli for more than one year and persist in excreting for decades thereafter (Table 1) [18–20]. Most chronic stool excretors had gallbladder disease and the female:male ratio was approximately 2:1 to 4:1 (Table 1) [10,18,19]. It became recognized that chronic urinary carriers also exist [18,21], as reported by investigators who performed repetitive cultures of convalescents’ urine over time (Table 1) [13,18,22]. Urinary carriers can also shed *S*. Typhi for many years or decades (Table 1) [13,18,19,23–27].

**Table 1:** Comparison of reported and unknown features of biliary versus urinary carriers.

	Biliary tract carriers	Urinary tract carriers
Main anatomic sites of carriage	Gallbladder [19]	Kidney [19,23–27,53]
Peak age	Middle-age adults [50,53,64]	Potentially second to fourth decades. Paucity of primary data to support an answer, based on pre-antibiotic era data [27]
Female:male ratio	Female > male [19,50,53,64]	Equal [19]
Clinical specimens tested to detect and monitor carriers	Bile and stool cultures [12,13]	Urine [13,21,23]
Typhoidal serovars reported	*S*. Typhi > *S*. Paratyphi A or B [50,64]	Paucity of primary data to support an answer
Level of excretion (colony forming units [CFU]/ml or CFU/gm)	10^6^ to 10^10^ [64,65]	Paucity of primary data to support an answer
Approximate % of acute carriers in the pre-antibiotic era who became chronic carriers	2%–5% of adult cases [18–20]	Paucity of primary data to support an answer
Duration of carriage from pre-antibiotic or early antibiotic eras	2–5 decades [18–20]	5–37 years [13,18,19,23–27]
Known predispositions	Prior gallstones [19]	Ureteral abnormalities; renal abscess; chronic cystitis; kidney stones, bladder stones [13,18,19,23–27]
Evidence of biofilm on recovered stones	Yes [50,52]	Paucity of primary data to support an answer
Associated with very high titers of serum Vi antibody	Yes [66,67]	Paucity of primary data to support an answer
Eradicable with antibiotics if strain is susceptible	Approximately 80% success [36]	Paucity of primary data to support an answer
Success of surgical intervention	Approximately 70%–80% cure with cholecystectomy alone [58,59,63,68–70]	If isolated to a single kidney, >95% cure with unilateral nephrectomy [24–27,71,72]
Role of point-of-care-ultrasound as an adjunct to screening for chronic carriers	May be helpful [73]	Paucity of primary data to support an answer

Prior to 1948, there was no specific treatment for typhoid fever and the case fatality rate was 10% to 20% [28,29]. Woodward and colleagues made a historic breakthrough when in 1948 they reported that chloramphenicol could markedly curtail the course of typhoid fever and convert a potentially fatal infection into a relatively short febrile illness [30]; however, 10% to 30% of cases treated with chloramphenicol experienced relapses of milder clinical typhoid that required another course of treatment [31]. From 1950 to 1971, when oral chloramphenicol was used worldwide and *S*. Typhi were almost universally susceptible, this antibiotic dropped the case fatality rate to approximately 1% and became a therapeutic intervention that controlled typhoid mortality globally. However, despite its efficacy in ameliorating the duration and severity of clinical typhoid, reducing complications, and diminishing case fatality, chloramphenicol did not prevent chronic carriage [29], nor could it terminate the chronic carrier state [31].

Following large epidemics of chloramphenicol-resistant typhoid fever in Latin America and Southeast Asia in the 1970s due to *S*. Typhi carrying an R factor plasmid encoding resistance genes [32], newer oral antibiotics shown to be efficacious in treating chloramphenicol-resistant infections, including trimethoprim/sulfamethoxazole and amoxicillin, became the new drugs of choice [32–34]. These new effective oral antibiotics did not prevent or reliably treat the carrier state [35]. In small clinical trials conducted in the late 1980s, oral fluoroquinolone antibiotics showed promising efficacy in curing the chronic carrier state [36,37]; one study reported a chronic carrier cure rate of 92% with a 28-day regimen of ciprofloxacin [36]. Since around 1990, *S*. Typhi circulating in South Asia, where most of the global burden of typhoid fever exists, has become multidrug resistant (MDR), and since 2016 extensively drug-resistant (XDR) *S*. Typhi strains have emerged and spread; the XDR strains offer few practical options for treatment [38,39].

The diminishing ability to treat typhoid in endemic areas and among travelers has renewed interest in controlling typhoid fever morbidity and mortality burden worldwide by preventing disease with vaccines, including World Health Organization–prequalified Vi conjugate vaccines, as well as water, sanitation, and hygiene (WASH) improvements. The most widely studied licensed Vi conjugate vaccine consists of *S*. Typhi Vi polysaccharide linked to tetanus toxoid as the carrier protein (Typbar TCV, Bharat Biotech) [40], which has been evaluated in post-licensure effectiveness trials in Nepal [41], Malawi [42], and Bangladesh [43]. Mass immunizations to assess the impact of administration of a single dose of this vaccine to control endemic and epidemic typhoid have been carried out in Pakistan [44], India [45], Zimbabwe [46], and Samoa [47]. Results from these extensive field assessments support widespread use of Vi conjugate vaccines in typhoid-endemic areas. Should the typhoid disease burden markedly decrease in previously hyperendemic areas following high levels of vaccination coverage (and possible herd effects), one upshot will be an increased interest in identifying and monitoring the chronic carriers within the population who will constitute the long-term reservoir of infection [48–51]. Some carriers may be amenable to treatment if their isolates are fluroquinolone-susceptible [36]. Other carriers will die off within the population over several decades, once amplified transmission has been reliably interrupted [48,50,51]. This evolving global epidemiologic situation has reawakened interest in the various types of chronic *S*. Typhi carriers that constitute the long-term reservoir.

Since the classic physiological niche for chronic *S*. Typhi infection is the gallbladder, most modern studies have understandably focused on biliary carriers, the role of cholelithiasis, and *S*. Typhi’s ability to create biofilms on gallstones [50,52]. The prevalence of chronic *S*. Typhi carriers in endemic populations parallels the prevalence of cholelithiasis and chronic gallbladder disease [50,53]. Thus, chronic carriage is several-fold higher in females than males, and in older adults (>40 years of age) than younger adults and teenagers (Table 1) [50,53].

This review focuses on the less appreciated, less frequent chronic *S*. Typhi carriers who harbor foci of infection outside the gallbladder, including the intra- and extrahepatic bile ducts and the urinary tract. Herein, we review chronic carriage in these other anatomic sites. Although much information presented is also relevant to *S*. Paratyphi A and B infections, we have limited our scope to *S*. Typhi as the main new specific intervention becoming available, Vi conjugate vaccines, applies only to prevention and control of typhoid fever.

## Methods

In assembling a database of literature to draw upon for this review, the following MeSH search terms were utilized: typhoid, persistent infection, coinfection, trematode infection, clonorchiasis, fascioliasis, opisthorchiasis, schistosomiasis, parasitic diseases, biliary tract disease, cholelithiasis, choledocholithiasis, pyelonephritis, nephrolithiasis, urolithiasis, and/or urinary tract infection. This primary database was cleaned to exclude literature without reference to *S*. Typhi. This secondary set of articles were then individually evaluated to see if their own references suggested yet uncompiled articles, including articles written in German, French, Spanish, Hindi, Japanese, and Chinese. Additional location of articles was performed via internet search of the National Library of Medicine, National Institutes of Health; the Health Sciences and Human Services Library, University of Maryland; Lane Medical Library, Stanford University; Cornell University Library; and HathiTrust Digital Library. Any unevaluated articles were added to the secondary database. All these were subsequently evaluated for their pertinence as primary literature regarding chronic *S*. Typhi carriage and any association with chronic *S*. Typhi carriage outside the gallbladder (chronic non-gallbladder hepatobiliary *S*. Typhi carriers, chronic urinary *S*. Typhi carriers, and chronic *S*. Typhi carriage associated with a parasitic coinfection). Articles were excluded if they did not represent primary literature or the information discussed did not provide primary information. However, any articles that referenced data presented whereby the primary article was unable to be produced was included.

### Chronic *S*. Typhi carriage outside the gallbladder

**Fig 1:**
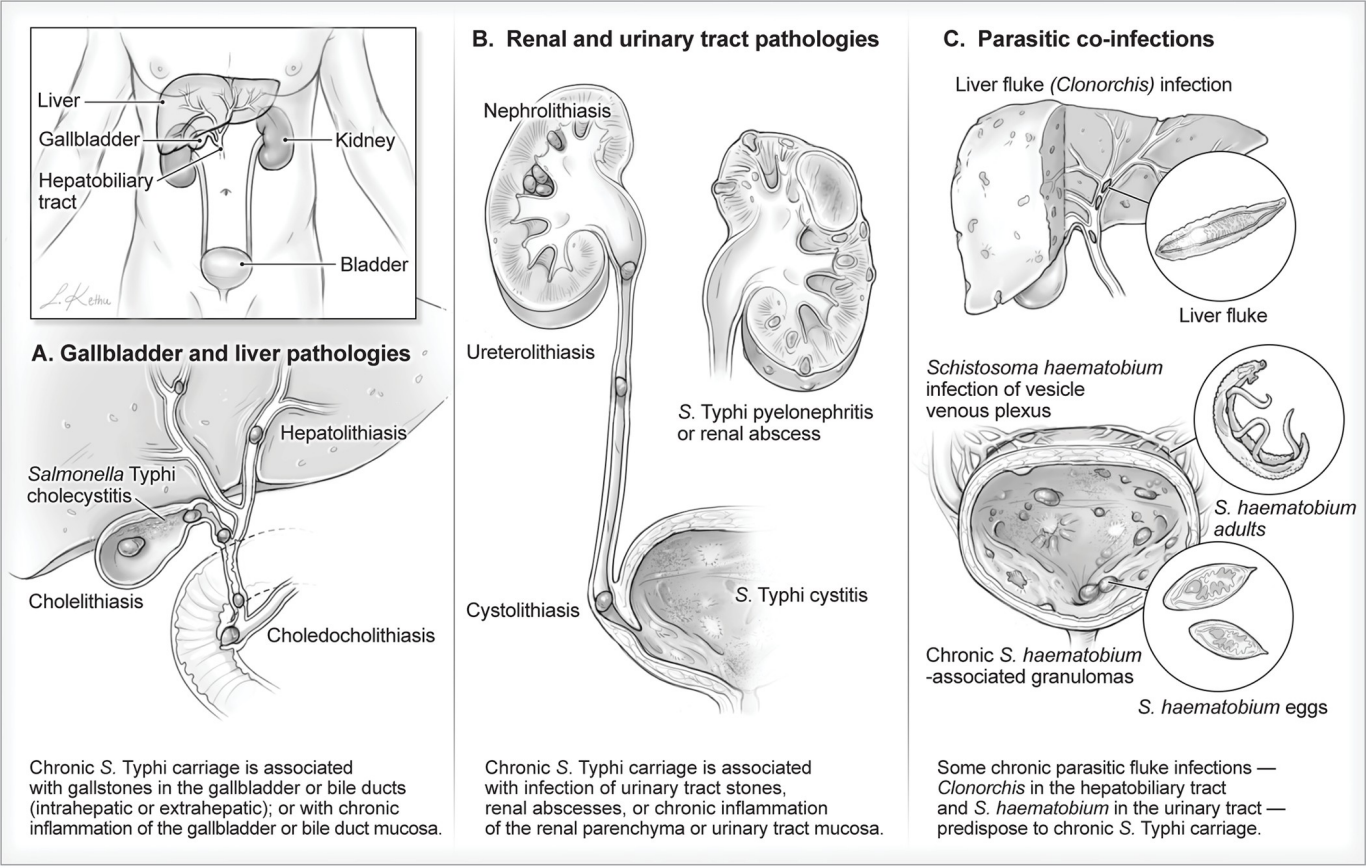
Anatomic locations and coinfections associated with chronic *Salmonella* Typhi carriage. Image credit: Lohitha Kethu, 2023.

There exist two categories of chronic *S*. Typhi carriers who are of special interest. These include chronic carriers who continue to shed *S*. Typhi in stool, despite undergoing cholecystectomy (Fig 1A), and chronic carriers who persistently shed *S*. Typhi in their urine rather than in their stools (Table 1) [13,18,19,21–23,26,31,49,54–63]. In both instances, the literature suggests a structural/inflammatory abnormality (e.g., gallstone, Fig 1A; or kidney stone, bladder stone, Fig 1B) versus the presence of a coinfection that provides a nidus for chronic carriage (Fig 1C). These “atypical” chronic carriers of *S*. Typhi also serve as long-term reservoirs of infection in the population who, when circumstances allow, transmit the infection to susceptible persons (Fig 1). These unusual varieties of chronic carriers must also be taken into account as programs are initiated to control typhoid fever and even to eliminate transmission in circumscribed populations within defined time periods.

### Chronic non-gallbladder hepatobiliary carriers

Given *S*. Typhi’s association with bile and gallstones, multiple authors have proposed that there may exist sites of chronic *S*. Typhi infection along the hepatobiliary tract in the absence of gallbladder infection per se. Cholecystectomy has been used to treat chronic typhoid carriers since at least 1907 [68], with a cure rate of approximately 70% to 80% [58,59,63,68–70]. In the pre-antibiotic era, when cholecystectomy was the only effective therapeutic option for terminating chronic excretion of *S*. Typhi by gallbladder carriers [19,22,68,69], various writers described instances in which *S*. Typhi was persistently cultured from the bile or stool of patients without gallbladders [19,49]. Within the antibiotic era, Woodward and colleagues (1950) described a carrier who underwent cholecystectomy and prolonged chloramphenicol treatment without cure [31], and Tynes and colleagues (1962) reported their failure to eradicate the chronic *S*. Typhi carrier state using combined cholecystectomy and antimicrobials available at the time [58]. Of note, these attempts were prior to the introduction of more effective antimicrobial regimens to treat chronic carriers (if the strains were susceptible) [33,36]. It was presumed that the nidus of chronic infection in such cases resided in areas of scarring or inflammation along the hepatobiliary tract [58,70].

### Concomitant liver fluke infection

Chronic infection with the liver fluke *Clonorchis sinensis* may predispose to chronic hepatobiliary *S*. Typhi carriage [61]. Clonorchiasis is associated with cholelithiasis, cholecystitis, and cholangitis [74], all of which may alter the anatomy of the hepatobiliary tract and possibly promote the *S*. Typhi chronic carrier state. The global prevalence of chronic clonorchiasis, predominantly East Asia and South East Asia, overlies areas of the world heavily affected by typhoid [75,76]. Thus, persons with chronic *C*. *sinensis* infection may be at increased risk for becoming a chronic *S*. Typhi carrier following acute *S*. Typhi infection. Theoretically, infection with other liver flukes, such as *Fasciola* spp. or *Opisthorchis* spp., might also predispose to chronic *S*. Typhi carriage since, like *C*. *sinensis*, they are also associated with chronic hepatobiliary inflammation. However, this has not been conclusively demonstrated [74]. Future work is needed to elucidate these potential coinfection relationships, which may be relevant for control of typhoid fever and elimination campaigns in certain geographic regions where those liver flukes infections are prevalent.

### Potential modern approaches for diagnosis and treatment

Modern procedures like magnetic resonance cholangiopancreatography (MRCP) and endoscopic retrograde cholangiopancreatography (ERCP) may theoretically allow the identification of structural abnormalities that are promoting conditions favorable for establishment of a chronic carrier state and may allow repair of those anatomic predispositions (e.g., hepatolithiasis, choledocholithiasis, sites of obstruction amenable to intervention). To our knowledge, there have been no reports of the use of these new technologies with respect to chronic *S*. Typhi carriers. More importantly, these procedures are limited by lack of availability in many typhoid-endemic LMIC settings. However, they are available in HICs such as the USA, where approximately 20% of the 300 to 400 annual cases of typhoid fever represent indigenous transmission and >50% of outbreaks are linked to a chronic carrier in the home or one who handled food [77].

### Chronic urinary carriers

In the late 19th and early to mid-20th centuries, many reports described patients with chronic urinary excretion of *S*. Typhi, in some cases with detectable bacteria in their urine for many years [21,23,24,26,27,49,54–57,60,72]. Progression to a chronic urinary carrier state is usually associated with a lesion or focus of inflammation in the kidney or urine collecting system (e.g., renal or bladder stone). Supporting this hypothesis, multiple case reports describe or indicate cessation of the carrier state following nephrectomy of a diseased kidney [24–27,71,72] or treatment of a diseased bladder with “urinary antiseptics” (i.e., oral urotropin, sometimes combined with other agents, such as boric acid) [55]; in the early 20th century, cystoscopy was already being utilized to diagnose and plan these nephrectomies for cure, and United States Army Soldiers could be court martialed for refusing surgical intervention in the setting of the chronic *S*. Typhi carrier state [72]. These findings indicate an interesting possibility: Just as *S*. Typhi is able to develop a chronic carrier state on or within gallstones and other centers of inflammation along the hepatobiliary tract, typhoid bacilli might do the same on or within kidney stones and along the urinary tract. Since certain other bacteria form biofilms on kidney stones, research should pursue whether *S*. Typhi also can [78]. Of note, early works suggest there is no sex-based difference in cases of chronic urinary carriers [19] (Table 1), as compared to the greater frequency of female versus male chronic gallbladder carriers [19,50,53].

### *Typhoid–Schistosoma* urinary tract coinfection

Hsiao and colleagues (2016) reviewed in detail the literature supporting a putative symbiotic interplay between certain *Salmonella enterica* serovars (Typhi, Paratyphi A, B and rarely C, and Typhimurium) and human schistosomiasis [79]. Most common was *S*. Typhi and *S*. *haematobium* coinfection [79]. The pathological and immunomodulatory consequences of chronic schistosomiasis favor the progression of *S*. Typhi infection to the chronic carrier state in a putatively symbiotic association between the two pathogens.

Further investigation into whether *Schistosoma* species other than *S*. *haematobium* may predispose to a chronic *S*. Typhi carrier state is warranted.

### The importance of chronic carriers

In areas of low transmission of *S*. Typhi, both observational and mathematical modeling data demonstrate the role of asymptomatic chronic carriers in maintaining a reservoir for typhoid endemicity (via short-cycle transmission), until these chronic carriers ultimately die out from the population [50,51]. In planning large-scale interventions for typhoid control, including possible elimination in circumscribed populations [47], chronic carriers must be taken into account. Unfortunately, the diagnostic screening tests utilized, heretofore, to identify possible chronic carriers are suboptimally sensitive and specific, and none are point-of-care [52].

Efforts to control and perhaps even eliminate typhoid must take into account the role of different types of chronic *S*. Typhi carriers in maintaining typhoid endemicity and difficulties treating carriage due to drug-resistant *S*. Typhi [38,39]. Particular emphasis has been placed on the paucity of data and clinical understanding of chronic urinary carriers. To this end, historical and contemporary typhoid vaccine challenge models did monitor stool cultures but did not include urine culture in standard practice [80]. This review aims to sensitize clinicians, epidemiologists, modelers, and public health practitioners to the role of chronic *S*. Typhi carriers in maintaining a long-term reservoir of infection in communities and to the various types of carriers that exist beyond the classic gallbladder carrier.

**Key Learning Points**

- Whereas most chronic infection with *Salmonella* Typhi resides within the gallbladder, other locations of chronic infection also exist including the urinary tract and the biliary tree absent the gallbladder.
- Contemporary data on chronic typhoid infection outside of the gallbladder are sparse.
- The long-term importance of chronic typhoid carriers in maintaining typhoid endemicity should not be underestimated.

**Top Five Papers**

1. Niepraschk. Beitrag zur Kenntnis der Verbreitung des Typhus durch Dauerausscheider. Zeitschrift für Hygiene und Infectionskrankheiten. 1909;64: 454–476.
2. Browning CH, Coulthard HL, Cruickshank R, Guthrie KJ, Smith RP. Chronic Enteric Carriers and their Treatment. London: His Majesty’s Stationery Office; 1933. p. 1–80.
3. Bondy PK, Barnwell CH. Chronic Typhoid Pyonephrosis: Report of a Case. J Urol. 1947;57(4):642–650. doi:10.1016/S0022-5347(17)69683-6
4. Tynes BS, Utz JP. Factors influencing the cure of Salmonella carriers. Ann Intern Med. 1962;57:871–882. doi: 10.7326/0003-4819-57-6-871
5. McFadzean AJ, Ong GB. Intrahepatic typhoid carriers. Br Med J. 1966;1(5503):1567–1571. doi: 10.1136/bmj.1.5503.1567
